# Envisioning the use of online tests in assessing twenty-first century learning: a literature review

**DOI:** 10.1186/s41039-017-0055-7

**Published:** 2017-08-07

**Authors:** Bopelo Boitshwarelo, Alison Kay Reedy, Trevor Billany

**Affiliations:** Charles Darwin University, Ellengowan Dr, Casuarina, NT 0810 Australia

**Keywords:** Assessment, Online tests, Online quizzes, Multiple choice questions, Higher education, Twenty-first century learning, Formative assessment, Feedback, E-assessment

## Abstract

The digital world brings with it more and more opportunities to be innovative around assessment. With a variety of digital tools and the pervasive availability of information anywhere anytime, there is a tremendous capacity to creatively employ a diversity of assessment approaches to support and evaluate student learning in higher education. The challenge in a digital world is to harness the possibilities afforded by technology to drive and assess deep learning that prepares graduates for a changing and uncertain future. One widespread method of online assessment used in higher education is online tests. The increase in the use of online tests necessitates an investigation into their role in evaluating twenty-first century learning. This paper draws on the literature to explore the role of online tests in higher education, particularly their relationship to student learning in a digital and changing world, and the issues and challenges they present. We conclude that online tests, when used effectively, can be valuable in the assessment of twenty-first century learning and we synthesise the literature to extract principles for the optimisation of online tests in a digital age.

## Introduction

In recent times, there has been widespread interest from governments, industry, and educators in identifying a model of learning and assessment in higher education that meets the challenges of learning in the digital present and prepares students for an uncertain future (Kinash 2015). The term twenty-first century learning is widely used to encapsulate the idea that fundamental changes in the nature of learning and education have occurred in the twenty-first century as a consequence of rapidly changing technologies and globalisation (Kereluik, Mishra, Fahnoe, & Terry, 2013). Hence, different forms of assessment that are commensurate with the twenty-first century are needed.

**Fig. 1:**
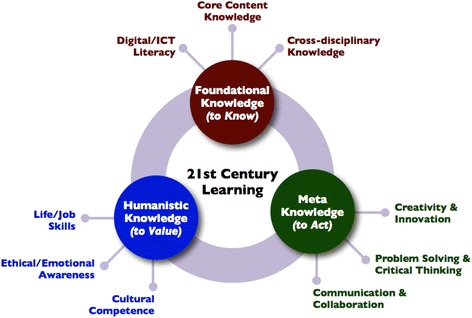
A framework of 21st Century Learning (Kereluik et al., 2013, p. 130)

Multiple and disparate interpretations exist as to what kind of knowledge and skills are needed to live and work in the twenty-first century, and hence, there is little clarity as to the forms of assessment that can be used most effectively to assess the knowledge and skills required for a digital age. For the purposes of this paper, our understanding of twenty-first century learning is based on the overarching framework of twenty-first century learning developed by Kereluik et al. (2013). Their synthesis of 15 widely used frameworks of twenty-first century knowledge produced “a coherent integrative framework” (Kereluik et al., 2013, p. 128) for conceptualising twenty-first century knowledge. This “framework of frameworks” (Kereluik et al., 2013, p. 129) contains three knowledge domains inherent in twenty-first century learning: foundational knowledge, meta-knowledge, and humanistic knowledge (see Fig. 1), with each domain containing three subcategories.

In addition, there are other approaches that contribute to our understanding of assessment in a digital age. Scott (2016) introduced the idea of “right” assessment within the context of flipping the curriculum or “FlipCurric”, where the focus is on considering assessment forms and practices that evaluate competencies and capabilities for the twenty-first century. Scott describes assessment for the digital age as being powerful, fit for purpose, valid, and focused on preparing graduates to be “work ready plus” (Scott, 2016), that is, ready to meet the challenges of current and future job markets. Assessment types such as problem-based learning, authentic learning tasks, and case studies feature highly as types of powerful assessment in that they evaluate students’ ability to consolidate learning across knowledge domains and to apply knowledge, skills, and capabilities that are relevant to living and working in a “volatile and rapidly transforming world” (Scott, 2016). These powerful forms of assessment align strongly with a constructivist approach to learning, which is the learning perspective most widely accepted in higher education as enhancing student learning (Anderson, 2016).

There has been a rapid growth in the use of online tests especially since the widespread implementation of LMS in higher education in the early part of the twenty-first century (Stone 2014). This trend, which is also evident at the authors’ institution, raises questions as to why online tests are being used so extensively, and whether their use aligns with the conceptualisation of twenty-first century learning and commensurate assessment practices. To respond to these questions, in this paper we review the literature to explore the current use of online tests in higher education, and particularly their relationship to student learning in a digital and changing world. We also identify issues associated with their use and propose principles to guide the use of online tests in the assessment of twenty-first century learning.

We use the term “online tests” to specify a particular type of ICT-based assessment, or e-assessment that can be used for diagnostic, formative, and summative purposes. While e-assessment can be used to broadly refer to any practice where technology is used to enhance or support assessment and feedback activities, online tests specifically refer to computer-assisted assessment where the deployment and marking is automated (Davies, 2010; Gipps, 2005). Online tests (also known as online quizzes^1^) are used extensively within learning management systems (LMS) in online and mixed mode delivery. For the purpose of this paper, online tests are distinguished from “online exams”, which are typically invigilated and conducted on computers in a controlled exam centre. The investigation of literature presented in this paper focuses on identifying the ways online tests are used and the extent to which their use supports the assessment of twenty-first century knowledge and skills.

## Methods

### Design of the literature review

We conducted a scoping literature review to identify academic papers and research reports that discuss the use of online tests in higher education. The review of literature focused on academic papers contained in English language journals using key search terms including *online assessment*, *online tests*, *online quizzes*, *multiple choice questions* and other related aspects, particularly in a higher education context. The search was done through Google Scholar and through our institution’s library databases. The review focused on literature published since the year 2000, which aligns with the widespread take up of the LMS by higher education institutions in the first few years of the twenty-first century, and reinforces the pivotal role the LMS has played in the digitisation of learning and assessment in higher education.

The literature search revealed over 50 relevant papers from publications (primarily scholarly journals) focused on general teaching and learning, educational technology or discipline-specific education (e.g. Advances in Physiology Education). The literature review was not discipline specific and online tests were identified that cross a range of disciplines, with the natural sciences and social sciences (including business and economics) quite highly represented, with arts and humanities less so. A significant number of the empirical studies reviewed were in specific discipline areas, including earth sciences, physiology, nursing, medical sciences/biology, psychology and business (for example, see Angus & Watson, 2009; Brady, 2005; Buckles & Siegfried, 2006; Smith, 2007; Yonker, 2011). The number of articles identified provided a large enough pool to gain insight into the use of online tests in higher education.

In the scan of literature, we identified only a few review studies related to online tests, three of which were in the broad field of e-assessment (Gipps, 2005; Stödberg, 2012; Sweeney et al., 2017) and one with a specific focus on feedback practices in online tests (Nicol & Macfarlane‐Dick 2006). The most recent of these three broad reviews (Sweeney et al., 2017) involved a systematic literature review of scholarly articles about technology-enhanced assessment published in the 3 years 2014 to 2016. This study looked at what technologies are being used in e-assessment and whether they are enhancing or transforming assessment practice; however, while it referred to e-assessment within the LMS, it did not refer specifically to online tests. The wide scope of the Sweeney et al. study contrasts with the targeted focus and review of case studies about feedback in online tests in the Nichol and McFarlane-Dick paper. The identification of so few review studies indicates the need for a synthesis of the disparate body of scholarship in relation to online tests such as presented in this paper.

## Results

### Exploring online tests

In this section, we review the literature around online tests to identify some of the research on their use in higher education contexts including the rationale for their use, their relationship to student learning, and trends in practice.

#### The rationale for using online tests

The use of e-assessment generally, and online tests in particular, has increased in higher education over the last two decades. This is a corollary of reduced resources for teaching and increased student numbers, meaning academics are required to do more with less while adapting to the increasing usage of technology in teaching (Donnelly, 2014; Nicol, 2007). The potential of technology has been harnessed to ameliorate the challenge of heavy academic workloads in teaching and for assessment, with the use of e-assessment providing a way to “avoid disjunction between teaching and assessment modes” (Gipps, 2005, p. 173). In other words, the growth in the use of ICTs as a mode of teaching necessitates their growth as a mode of assessment, which, Gipps’ claims, increases the mode validity of the assessment practices.

Additionally, Gipps (2005) also points to efficiency and pedagogic reasons for using online tests. Because of the automated marking and feedback, online tests are viewed as highly efficient, fast and reliable, making them especially useful where large numbers of students are concerned. Consequently, online tests are very common with large first year classes. Their efficiency also emanates from the ability to test a wide range of topics in one test in a short duration as compared to assessment where responses need to be constructed (Brady, 2005). The capability to create, manage, and deploy online tests within an LMS means that a lot of previously manual work is automated (Pifia, 2013). To add to the efficiency, most major textbook publishers, such as Cengage Learning, Pearson Education and McGraw-Hill Education, have linked online question banks to their textbooks, at least in disciplines where the use of online tests is common which easily integrates with the more popular LMSs such as Blackboard™. Instead of academics creating questions from scratch, they can instead select or import them wholesale from these test banks.

While the mode validity and efficiency reasons for using online tests are easily observable, it is the pedagogic reasons for using online tests that are undoubtedly the most critical. It is imperative to unpack whether indeed online tests support and assess student learning in higher education, and if so, what kind of learning they facilitate and in what circumstances. The next few sections explore the literature around these questions.

#### Cognitive levels of questions

Typically, online tests involve the use of multiple choice questions (MCQs), true/false questions, matching questions as well as predetermined short answer questions. LMSs provide opportunities for these and other different types of questions to be included in the creation and deployment of online tests. Of these, MCQs are the most commonly used question type (Davies, 2010; Nicol, 2007; Simkin & Kuechler, 2005). Hence, the discussion will primarily, but not exclusively, focus on them.

The focus of MCQs can vary from recall type questions to questions that demand higher cognitive levels of engagement (Douglas et al, 2012). Therefore, MCQs can be used to assess different types of learning outcomes. For example, a two-factor study between study approach and performance (Yonker, 2011) made a distinction between factual MCQs and application MCQs. The distinction was a reflection of the level of difficulty or cognitive demand of the questions as shown by the fact, in this study, that students who employed surface learning approaches were found to have performed relatively poorly in application MCQs as compared to those who used deep learning approaches. Using Bloom’s taxonomy, some authors have asserted that MCQs are most suitable for the first three cognitive levels of remember, comprehend and apply (Simkin & Kuechler 2005; Douglas et al, 2012) and, to some extent, the level of analysis (Buckles & Siegfried, 2006; Brady, 2005).

#### Online tests in context

The effectiveness of online tests can be realised when they are implemented in the context of the whole learning experience, including using other assessment types. Indeed, Douglas (2012) recommends that online tests can be used to full effect when used in conjunction with other forms of assessment. Smith (2005), in a study that investigated how performance on formative assessment (including online tests) related to learning as assessed by final examination, concluded that frequent and diverse assessment can enhance student engagement and performance. Essentially, each assessment type in a suite of assessment approaches in a particular unit of study, including online tests, should target appropriate learning outcomes and complement other types of assessment. Furthermore, a study by Donnelly (2014) found that case-study-based MCQs led to a higher level of learning and deeper processing of information over MCQs not based on case studies. This led the author to conclude that blending of assessment methods (in this case MCQs and case studies) can lead to enhanced student learning while also addressing challenges of large class sizes. This is consistent with Nicol (2007), who concludes if MCQs are designed creatively and the context of their implementation is managed accordingly, they can be used to achieve the *Seven Principles of Good Feedback Practice* (Nicol & Macfarlane-Dick, 2006) including clarifying good performance, self-assessment and reflection, and dialogue.

#### Online tests and formative learning

The literature points to online tests being best suited for formative purposes, that is, as assessment for learning. In particular, the studies reveal nuances in the relationship between online formative tests and student learning. Pedagogic reasons for using online tests include the opportunities they provide for automated rich, descriptive, formative feedback which potentially scaffolds the learning process and allows learners to self-evaluate and improve their performance in preparation for summative assessment (Gipps, 2005; Nicol, 2007). Formative online tests can also be used for diagnostic purposes and assisting staff to identify where they should focus their teaching efforts.

Formative online tests contribute to student learning, as measured by summative assessment and particularly examinations (Angus & Watson, 2009; Kibble, 2007; Smith, 2007). This is particularly the case if the same types of outcomes or cognitive abilities are assessed by both the formative tests and the summative assessment (Simkin & Kuechler, 2005). The positive correlation between online formative tests and student learning (as indicated by summative achievement) is enhanced by task design that includes the following specific features.

Firstly, in a study using statistical analysis to compare formative scores to summative scores, Smith (2007) found that increased student learning takes place where there is engaged participation in online tests, which can be encouraged by assigning credit/marks to them.

Secondly, studies using statistical analysis (Angus & Watson, 2009; Smith, 2007) and those which combine statistical analysis with mixed method surveys on student and staff perceptions on the role of online formative tests (Kibble, 2007) show that student learning is enhanced where the online tests are regular and of low stakes credit and not too overwhelming.

Thirdly, surveys of staff and/or students perceptions of online tests (Baleni, 2015; Kibble, 2007) identified student learning is enhanced where multiple attempts (at least two) at a test are available, with qualitative feedback given after each attempt. The multiple attempts not only provide an opportunity for feedback and revision of material but they also play a role in building confidence in taking the online tests and subsequent exams.

#### The nature of feedback

The nature of feedback practice is critical in facilitating the learning process. While online tests are commonly set up to give feedback about the correctness of the student’s response to the question, some of the scholarly articles reviewed reported on improved student performance when immediate corrective feedback, or feedback about how to improve performance, was built into the test (Epstein, Lazarus, Calvano, & Matthews, 2002; Gipps, 2005; Kibble, 2007). This corrective feedback could include referring students to a particular module or page in the text for further study.

Feedback can be in the form of quantitative feedback to inform students about their grades and qualitative feedback that allows students to review their understanding of the content. For example, Voelkel (2013) describes a two-stage approach to online tests where the first stage is characterised by a formative test in which prompt qualitative feedback is provided to students. During this stage, students have multiple attempts to achieve at least 80%, and once this is achieved they are given access to the second stage of the test. The second stage is summative in nature and contains variants of the questions used in the first stage. This staged use of online tests reportedly improves performance not only of good students but also of weak ones.

Beyond automated immediate feedback and multiple attempts, Nicol (2007) presents case studies of good feedback practices in online tests (Nicol & Macfarlane‐Dick, 2006). The practices identified include the staging of online tests across the semester to facilitate provision of feedback to students as well as diagnostic feedback to the lecturer to inform teaching (Bull & Danson, 2004); facilitating reflection/self-assessment and enhancing motivation to engage students more deeply with test questions (and answers) through confidence-based marking (Gardner-Medwin, 2006); and using online MCQs and electronic voting systems/polling and subsequent peer interactions to facilitate reflection/self-assessment and dialogue (Boyle & Nicol, 2003; Nicol & Boyle, 2003). The major emphasis of Nicol’s (2007) analysis of these cases was to highlight how MCQs can be used to good effect to enhance learner self-regulation.

#### Student attitudes to online tests

While a positive correlation generally exists between formative tasks and summative performance, student learning is also dependent on factors that are not directly related to assessment but which nonetheless have a direct bearing on students’ participation and engagement with formative assessment. For example, formative assessment will only benefit students who are motivated to achieve high performance (Kibble, 2007; Smith, 2007) and make an effort to engage and learn (Baleni, 2015). Students who do not engage, either because of constraining circumstances such as being time poor or as a result of a lack of interest in excelling, are unlikely to significantly benefit from formative assessment tasks (Smith, 2007).

While most of the studies reviewed focused on comparative analysis between engagement with formative online tests and summative performance, a few studies also investigated student and staff perceptions of online tests. These studies generally revealed a positive attitude towards MCQs by both staff and students (Baleni, 2015; Donnelly, 2014; Kibble, 2007). The reasons for students’ positive attitudes towards online tests are varied but seemed to be mostly ascribed to the perceived easiness of MCQs (Donnelly, 2014; Gipps, 2005). In addition, some students liked the idea of multiple attempts and feedback (Baleni, 2015; Kibble, 2007). Other students thought that having a choice of answers in MCQs assisted their memory and thinking process (Donnelly, 2014). The convenience of being able to take online tests anywhere was also a favourable factor for students (Baleni, 2015). However, on the negative side, a reason that students gave for not liking MCQs was that this form of assessment did not allow them to demonstrate their level of knowledge (Donnelly, 2014).

#### Online tests and twenty-first century learning

Overall, the literature reviewed reveals that online tests should be fit for purpose, assess appropriate learning outcomes and be used in conjunction with other forms of formative and summative assessment targeting different types of outcomes if they are to effectively lead to student learning (Brady, 2005; Smith, 2007; Yonker, 2011). Online tests should be used strategically to facilitate learner engagement and self-regulation. Online tests are used predominantly to assess the foundational knowledge domain; however, with some creative thought, effort, time, and appropriate tools within and outside the LMS, online tests could be applied to the other twenty-first century learning domains of humanistic knowledge and meta-knowledge as well as to achieve the concept of powerful assessment (Scott, 2016).

Examples from the literature show ways in which online tests can be used in the assessment of twenty-first century learning. When designed and used effectively, they can assist academic staff teach large student cohorts and provide students with immediate and corrective feedback that can enhance subsequent performance. Yet while online test clearly have a specific and important role in the assessment of student learning in higher education, they are not without challenges. The following section reviews some of those challenges and issues associated with using online tests.

## Discussion

### Challenges and issues around using online tests

A number of challenges and issues are commonly raised in the literature concerning online tests. These include cheating by students (Arnold, 2016; Fontaine, 2012); concern that online tests largely test only the lower levels of comprehension (McAllister & Guidice, 2012); an increased dependency on data banks of MCQs developed and provided by text book publishers (Masters, 2001); over or under testing based on the frequency of online tests; and the inflexibility of online tests to cater for diverse groups of students (Stupans, 2006).

#### Cheating

The literature indicates numerous practices that students engage in while doing online tests that are often considered to be cheating. This includes students treating them as open book tests, which may involve using multiple computers for fast searching for answers (Fontaine, 2012). If not explicitly stated otherwise, students may consider the practice of online searching during an online test as acceptable, indeed resourceful. On a more serious level, in online tests there is an increased possibility of students using a proxy to complete the test or of colluding to do them in small groups. Another issue is multiple people logging in under the same username at the same time on different computers to help each other take the test. In order to counter some of these practices, e-proctoring systems that monitor students visually and digitally while they are in the process of doing an online test are available and used increasingly by higher education institutions. However, regardless of their use, online tests remain susceptible to some of these practices.

Studies are inconclusive as to whether there is an increase in cheating in online courses as opposed to face-to-face (f2f) courses (Harmon, Lambrinos, & Buffolino, 2010) but they do show that cheating risk is higher in unproctored online assessments. Cheating in low- or zero-value unproctored online tests raises different levels of concern for different lecturers, but it has been shown (Arnold, 2016) that cheating in formative online unproctored tests does not pay off in the long run as those students are likely to perform worse in final summative assessments than students who have not cheated.

Cheating is often deterred by utilizing control features that may be built into the online test delivery software in an LMS, for example, randomization of questions and responses, single question delivery on each screen, no backtracking to previous questions, and/or setting very tight time frames in which to answer the questions. One survey of students (Harmon et al., 2010) concerning tactics to deter cheating rated the following as the top 4, in order of effectiveness: using multiple versions of a test so that students do not all receive the same questions, randomizing question order and response order, not using identical questions from previous semesters, and proctor vigilance.

Harmon et al. (2010) provide a warning to higher education institutions that do not address issues of cheating in online tests. They point out that higher education institutions that are “tone deaf to the issue of proctoring online multiple choice assessments may understandably find other institutions reluctant to accept these courses for transfer credit” (Harmon et al., 2010, Summary para.2).

An additional and emerging threat to online tests, as well as to other forms of e-assessment, is their susceptibility to emerging cyber security threats such as hacking. Dawson (2016) identifies this as a particular concern in the context of invigilated online exams conducted on students’ own devices. While this threat cannot be disregarded for online tests, hacking and other cyber security threats are more likely to impact high-stake examinations and be less of a concern for low-stake formative assessment of the type that is more usual in online tests.

#### Feedback and learning

Although the provision of immediate feedback is a positive feature that can be enabled in online tests, this feature is often disabled to reduce the opportunity for cheating in online tests that are used for summative purposes. Lack of feedback can have negative memorial consequences on student learning particularly when MCQs are used. MCQs expose students to answers that are incorrect and this can reinforce incorrect understandings and influence students to learn false facts if feedback is not given (Fazio, Agarwal, Marsh & Roediger, 2010; Roediger & Marsh, 2005). This negative impact of MCQs is reduced when immediate or delayed feedback is provided (Butler & Roediger, 2008).

#### Targeting low cognitive levels

While online tests can be used to assess learning at a range of cognitive levels (McAllister & Guidice, 2012), they generally are only used to assess low-level cognition. There is evidence to indicate, “multiple-choice testing all too frequently does not incorporate, encourage, or evaluate higher-level cognitive processes and skills” (McAllister & Guidice, 2012, p.194). The impact of this on student learning will be dependent on the level and learning outcomes for the unit, as well as the weighting of the assessment and the mix of assessment types being used.

In previous years, before the advent of widespread use of the online medium, it was not uncommon to have textbook questions that were shallow. For example, Hampton (1993) found that 85% of MCQs and true/false format questions provided by a textbook publisher were aimed at remembering and recalling facts. This is no different from current online test banks. However, as previously mentioned, depending on the underpinning pedagogical principles and their context, online tests can be used to facilitate higher-order learning, for instance through the use of case-study-based MCQs (Donnelly, 2014; Hemming, 2010).

Multiple choice questions are also known to encourage guessing by students (Douglas, Wilson, & Ennis, 2012) and the scoring systems in LMSs may not be able to adequately cope with negative scoring techniques which provide a means to statistically counteract guessing. Furthermore, in formative assessment, students are often not inclined to find the correct answers and the reasoning behind them for questions that they answered incorrectly. In summative assessment, students often use a pragmatic approach to gaining the score that they need, taking a strategic rather than a deep approach to learning through assessment (Douglas et al., 2012). These factors contribute to the concerns of academic staff about whether the use of online tests represents good practice in assessment design (Bennett et al., 2017).

#### Publishers’ test banks

Many textbook publishers provide banks of test questions that can be deployed as online tests through institutional LMSs. The influence that profit-driven publishers have in dictating assessment practices in higher education raises concerns for many, particularly about the quality of the assessment questions and the suitability of MCQ format assessment in testing twenty-first century skills and knowledge (Vista & Care, 2017). The cognitive level of the questions in publisher test banks is often at the recall level, as discussed above. Additionally, there is often little security around the storage of test bank questions, and students usually have access to the same publisher test bank questions as academic staff when they purchase the textbook. While this provides an opportunity for students to access and practice test bank questions at their leisure, it can raise concerns for academic staff if they do not want students to see questions that may form part of graded assessment.

A highly publicised example of students having access to a publisher’s test bank questions was in 2010 at the University of Central Florida (Good, 2010) when approximately 200 students admitted to having access to the test bank prior to a mid-term online test. Much of the discussion around this case centred around whether the students were using justifiable resources for revision purposes and whether the publisher’s questions should only be used for formative purposes, implying that the lecturer should have written the summative test questions himself but did not and was therefore, in some way, negligent.

An additional problem with publishers’ test banks is that the evaluation of questions in them has revealed that they may not always be as rigorously assessed for reliability and validity as would be necessary for summative assessment (Masters, 2001). Ibbett and Wheldon (2016)) identify a number of flaws that are associated with construction of MCQs, particularly those sourced from publishers’ test banks. They contend that one of the serious flaws in these test banks relates to clueing signals in questions, which increase the possibility of students guessing the correct answers. In a review of a sample of six well-established accounting textbooks, they found that at least two thirds of the questions had some clueing signals. Their findings point to a greater need for scrutiny of questions from publishers’ test banks. Indeed, while lecturers expect that questions contained in publishers’ test banks will all function correctly, are error-free and are well written, this is not always the case.

There are also issues around the cost and accessibility of publishers’ test banks. As a security measure, some online publisher tests can only be accessed in versions of the textbook that include a specific code. That is a challenge for the lecturer if the cohort includes students who do not purchase that version (Bennett et al., 2017). There is strong evidence of high levels of financial stress on higher education students, particularly those from low socioeconomic backgrounds (Karimshah, 2013). The use of highly protected publisher test banks can disproportionately impact on these students.

#### Frequency

Running regular low-stake (for example, weekly) online tests is a popular approach (Bennett et al., 2017) designed to prevent students from falling behind. The provision of practice online tests, which are not graded, is common as a method to familiarise students with the functionality and requirements of summative tests. It is generally found that the number of students attempting the practice tests reduces as the unit progresses (Lowe, 2015) but those that do complete the practice tests tend to perform better in the summative tests.

While regular online tests and multiple attempts are recommended, there is a risk that a high frequency of tests can become overwhelming for both staff and students. Therefore, a balance needs to be struck between optimising student learning and consideration for staff and student workload.

#### Student diversity

Twenty-first century learning takes place in a globalised world, where university classes are characterised by cultural as well as demographic diversity (Arkoudis, 2014). Students’ views of assessment and their understanding of the purpose of assessment are related to their culturally linked experiences of education (Wong, 2004). Students’ differing knowledge, skills, and confidence in using digital technologies may also impact on assessment outcomes and where online tests are used, consideration needs to be given to preparing students so they are not disadvantaged by the technology or procedures used (Stödberg, 2012). This is particularly the case for older students, indigenous students and international students from certain countries, who may not have current or strong knowledge of digital technologies used in higher education. There is, however, little in the literature reviewed that addresses the use of online tests with diverse student cohorts.

The defined and tight time frames set for online tests, often used to deter cheating, can cause problems for students with a slow reading speed, which may include students for whom the language of instruction is not their first language. As a consequence, questions used in online tests do need to be reviewed for whether they assess the topic knowledge or whether they are testing high levels of language proficiency as well as the topic knowledge (Stupans, 2006).

An increasing number of students from non-traditional backgrounds are entering into higher education, not as direct school leavers but entering or returning to tertiary study later in life. Yonker (2011) shows that older students tend to perform better in online tests irrespective of whether they are based on factual or applied knowledge. The implication here is less to do with the design of online tests but relates more to analysing student cohort demographics and adapting the teaching strategies accordingly. This includes diagnosing problem areas for a specific group and giving targeted feedback.

### A vision for online tests in a digital future

Online tests have an immense potential to play an important educative role in student learning in higher education despite the challenges and issues associated with their use. However, given the range of assessment options available, and particularly given the emphasis on authentic forms of assessment in Scott’s vision of “right” assessment for twenty-first century learning, the use of online tests needs to be considered carefully to ensure that they are fit for purpose. Decisions about when and how to use online tests in higher education are important questions that need to take into account a range of factors including the benefits for both students and academics.

In this section, we utilise the Assessment Design Decisions Framework (Bearman et al., 2014, 2016) to guide assessment decision making around the use of online tests. The framework can assist curriculum developers, learning designers and academics to systematically work through key questions in determining the type of assessment to be applied. The Assessment Design Decisions Framework breaks the decision-making process around assessment into six parts: purposes of assessment, context of assessment, learner outcomes, tasks, feedback processes, and interactions. The first three parts of the Assessment Decisions Design Framework emphasise the considerations that take place in determining the assessment type. These include clarifying the purpose of the assessment and the context in which it takes place, as well as determining how the assessment task aligns with unit and the course level outcomes. The third and fourth parts of the framework relate to aspects of the task design itself and feedback processes built into the task design. Finally, the sixth part relates to interactions between all stakeholders about the assessment that are integral to its review and continual improvement.

In Table 1, we have synthesised the literature around online tests and present principles to maximise the use of online tests in evaluating twenty-first century learning. The framing of the principles against each stage of the Assessment Decisions Design Framework provides a systematic approach to considering the use of online tests at each stage of the assessment decision-making process.

**Table 1:** Principles for the design of online tests

Stages of the Assessment Design Decisions Framework	Principles for the design of online tests
Purposes of assessment • Support student learning? • Generate grades that will form part of subsequent certification? • Equip learners for making future judgements?	Online tests can be used effectively for the following purposes: • Supporting student learning through the use of low stakes, formative assessment • Deepening student learning by integrating them with authentic or communicative tasks, thus contributing to real-life decision making and problem solving • Assessing understanding of a breadth of content
Context • Characteristics of learners/students • Institutional assessment principles and policies • Professional, vocational or employment-related requirements • Departmental, disciplinary and personal norms, expectations and ideas • The overall program and the role of the unit module • Learning environment e.g. mode (online/face-to-face/blended); class size	Online tests are best used in the following context: • Managing resource constraints with large student cohorts • Online and blended learning environments • Student cohorts who are motivated to engage
Learner outcomes Consider the following: • Unit/module learning outcomes? • Overall program learning outcomes? • Professional requirements? • Learners’ general professional or intellectual development?	Learner outcomes are best achieved when online tests: • Are clearly aligned with specified unit learning outcomes • Form a basis for the achievement of higher-order learning outcomes and professional requirements • Form part of an integrated and holistic teaching and assessment approach in a unit • Support curriculum sequencing
Tasks • What kinds of tasks do learners need to engage in to: a) develop and b) demonstrate their learning? • What is the rationale for each task? • How do the tasks drive learning? What do the tasks specifically require learners to do? • What will be the criteria for successful completion? • How are tasks best distributed across the semester including their relationship with other tasks within the unit and within the program? • How will learners contribute to assessment processes? • Which tasks will be graded?	The design of online test is most effective when: • They are used regularly • They are used primarily for formative purposes • Assessment is of foundational knowledge • They are used for self-assessment and contribute to self-regulation and deep learning • The frequency and sequencing of formative online tests is synchronised and integrated with other learning, assessment and feedback activities • They are low stakes • They allow for multiple attempts • Students are encouraged to achieve a threshold mark in formative online tests prior to doing a summative test • Test questions are valid and contextually relevant • The question types is fit for purpose • Publisher-supplied test bank questions are reviewed for suitability against the purpose and learning outcomes • They stimulate peer interactions • The issue of cheating is irrelevant • Strategies are used to minimise the probability of cheating, particularly in medium to high stakes summative online tests e.g. limiting test duration, randomising test items and responses, proctoring and lockdown browsers
Feedback processes • How will tasks be located, related to each other and staged to produce multiple feedback opportunities? • What types of feedback information will be provided and by whom? • How will learner performance be used to influence the (re)design of later tasks?	Effective feedback to students: • Is immediate • Is corrective in addition to communicating correctness • Refers students to relevant resources to reinforce learning • Enhances learner self-regulation and motivation • Builds confidence and optimises performance • Encourages deep learning • Synchronises and integrates with learning activities • Enhances student achievement in future assessments Feedback about test performance should be as follows: • Sourced and analysed • Used to inform teaching • Used to improve question and overall test design
Interactions • How will resistance or engagement from learners or colleagues influence assessment processes? • How will learners understand what is required in the assessment task(s)? • What information will be needed to improve this assessment for subsequent occasions? • What associated changes in teaching and learning activities will be required?	Online tests are enhanced by the following: • Making the purpose and instructions for the use of online tests explicit for students and all teaching staff in a unit • A continuous improvement process which involves input from students and all teaching staff in a unit • A continuous process of experimentation, reflection and sharing of practices with colleagues and others

The principles articulated in Table 1 provide a systematic approach for academic staff and educational designers to make decisions about whether to use online tests as part of their assessment strategy in a particular unit. They also provide a guide for the design, development and deployment of online tests that enhance student learning as well as evaluate it. As a word of caution, these principles have not yet been applied to practical, real-world decision making about online tests. They have been developed as the first stage of a study exploring the widespread use of online tests at the authors’ institution. We anticipate that the principles will be refined as they are applied and evaluated as part of the study.

## Conclusion

Our review of the literature indicates that while online tests are often poorly designed and are predominantly used in the assessment of low-level thinking, they can be used effectively to assess twenty-first century learning, particularly but not exclusively in the foundational knowledge domain. Online tests can be designed to align with the concept of powerful assessment through selecting the format of the questions, the cognitive level of the questions, and the philosophical approach embedded in the task design that are fit for purpose and focused on authentic contexts. For example, this may be through using a case study approach, targeting cognitive engagement beyond the level of recall, and providing opportunities for group or peer learning around the online test to align with a constructivist philosophy.

The literature points to the ways in which online tests can enhance and even transform assessment through effective, innovative and creative use of their inherent features such as immediate feedback and scalability. In so doing there is clear evidence that online tests can be used to counteract high workloads of academics and particularly in the assessment and marking of large student groups, while providing students with immediate and quality feedback that contributes to their learning. The literature also indicates some of the challenges involved in the use of online tests such as the widespread use of publisher test banks focused on euro-centric knowledge contexts and the overrepresentation of online test questions that assess low levels of cognition. These challenges lead us not to dismiss the value of online tests in the assessment of twenty-first century learning but to identify how these concerns can be addressed.

In conducting the review, we also found significant gaps in the literature around online tests that point to the need for investigation. While online tests are mainly used in the assessment of foundational knowledge, there is some evidence in the literature around the use of online tests in the assessment of the meta-knowledge domains of twenty-first century learning but almost none in assessing humanistic knowledge. This may be a result of the limited use of online tests in the humanities. There are also gaps in understanding the experiences of students from diverse linguistic and cultural backgrounds in online tests. This area of research is particularly needed given the internationalisation of higher education in terms of curricula and student mobility yet the extensive reliance of Anglo-centric publishers’ test banks in the development of online tests.

In conclusion, we have drawn from the literature on online tests to distil a set of initial principles for decision making in relation to the selection and design of online tests in the context of twenty-first century learning. This study concludes that the limitations that are evident in the use of online tests in the digital present are not inherent features of online tests, but are a product of poorly conceived design, development, and deployment of online tests. Using the principles to guide strategic decision making about online test, we envision a digital future where online tests are used when they are fit for purpose and are optimised for the assessment of and for twenty-first century learning.

## Abbreviations

ICT: Information and communications technology
LMS: Learning management system
MCQ: Multiple choice question

## Footnotes

1 The term online tests is preferred because it is more encompassing
